# Gut microbiota alterations in primary biliary cholangitis: a systematic review and meta-analysis

**DOI:** 10.3389/fmicb.2026.1847865

**Published:** 2026-06-10

**Authors:** Ren Qianlang, Zhao Yanxin, Xiong Dejia, Wang Guoqing, Lu Lihong, Chen Hang, Wang Xianmei, Lv Junyan, Ma Lanqing

**Affiliations:** Yunnan Institute of Digestive Disease, The First Affiliated Hospital of Kunming Medical University, Kunming, Yunnan, China

**Keywords:** alpha diversity, dysbiosis, gut microbiota, meta-analysis, primary biliary cholangitis, systematic review

## Abstract

**Background:**

Primary biliary cholangitis (PBC) is a chronic autoimmune cholestatic liver disease. Increasing evidence suggests that gut microbiota dysbiosis may contribute to its pathogenesis and progression. However, existing studies are limited by small sample sizes, methodological inconsistency, and the lack of quantitative synthesis, resulting in insufficient consolidated evidence.

**Objective:**

This systematic review and meta-analysis aimed to evaluate alterations in the gut microbiota of patients with PBC.

**Methods:**

PubMed, Web of Science, Embase, and the Cochrane Library were systematically searched from inception to January 23, 2026. Outcomes included alpha-diversity indices, beta diversity, and taxonomic alterations. Meta-analyses were performed using standardized mean differences (SMDs) with 95% confidence intervals (CIs). Sensitivity analysis, subgroup analysis, and meta-regression were conducted to explore potential sources of heterogeneity.

**Results:**

Of 1,326 records screened, 10 studies involving 1,057 participants (607 patients with PBC and 450 controls) were included, and all studies had Newcastle–Ottawa Scale scores of at least 7. Compared with controls, patients with PBC had a significantly lower Shannon index (SMD = −0.72, 95% CI: −1.22 to −0.22, *p* < 0.001; I^2^ = 91.0%) and fewer operational taxonomic units (OTUs) (SMD = −0.57, 95% CI: −0.81 to −0.33, *p* < 0.001; I^2^ = 45.4%), whereas the Simpson index was significantly higher (SMD = 0.81, 95% CI: 0.40 to 1.21, *p* < 0.001; I^2^ = 44.4%). After exclusion of an outlier study, the Chao1 index was also significantly reduced (SMD = −0.38, 95% CI: −0.63 to −0.15, p < 0.001; I^2^ = 22.6%), while no significant difference was observed for the ACE index. Most studies also reported significant differences in beta diversity between patients with PBC and healthy controls. Taxonomic analysis showed enrichment of *Neisseria*, *Klebsiella*, *Veillonella*, *Bifidobacterium*, *Lactobacillus*, *Streptococcus*, *Enterococcus*, *Clostridium*, and *Escherichia*, whereas *Bacteroides*, *Faecalibacterium*, *Blautia*, *Roseburia*, *Coprococcus*, *Oscillospira*, and *Morganella* were generally depleted in PBC. Meta-regression did not identify age, geographic region, or sequencing platform as significant sources of heterogeneity.

**Conclusion:**

PBC is associated with gut microbiota alterations, characterized by reduced alpha diversity, altered community structure, and taxonomic remodeling. Longitudinal and functional studies are needed to clarify causality, mechanisms, and clinical applications.

## 1 Introduction

Primary biliary cholangitis (PBC), formerly known as primary biliary cirrhosis, is characterized by immune-mediated, non-suppurative destructive cholangitis involving the small intrahepatic bile ducts (Tanaka et al., 2024). PBC is distributed worldwide and can affect diverse populations, although it occurs predominantly in middle-aged and older women. Recent evidence suggests that the gap in disease prevalence between women and men may be narrowing compared with previous estimates (Chinese Society of Hepatology, 2022; Tan et al., 2026). The global annual incidence of PBC is approximately 1.8 per 100,000 persons, with a prevalence of 18.1 per 100,000. However, marked regional differences have been reported. Prevalence is highest in the Americas, followed by Europe and the Western Pacific region, at approximately 28.5, 18.6, and 9.9 per 100,000 persons, respectively. Incidence shows a similar pattern, being higher in the Americas than in the Western Pacific region. Between 2010 and 2020, the Western Pacific region showed the fastest increase in prevalence, whereas Europe showed a decreasing trend (Tan et al., 2026). The typical clinical manifestations of PBC include fatigue and pruritus. At least 50% of patients with early-stage PBC are asymptomatic, and the severity of pruritus appears to be unrelated to disease stage or activity. In advanced cases, PBC may progress to cirrhosis, liver failure, and hepatocellular carcinoma (Natarajan et al., 2021; Tana and Hirschfield, 2026). The pathogenesis of PBC is complex and is generally considered to result from the interplay of genetic susceptibility, environmental triggers, and immune dysregulation (Ronca et al., 2025). Ursodeoxycholic acid (UDCA) remains the only first-line therapy currently available; however, approximately one-third of patients show an inadequate response to UDCA, and most second-line therapies are still under development (Han et al., 2024). Therefore, further elucidation of the pathogenic mechanisms underlying PBC is of substantial clinical and scientific importance.

In recent years, increasing attention has been directed toward the role of the gut microbiota in the pathogenesis of PBC. A growing body of evidence indicates that patients with PBC exhibit marked gut microbial dysbiosis (Tang et al., 2018; Furukawa et al., 2020; Liu Q. Y. et al., 2025). Multi-omics studies have demonstrated reduced microbial diversity in PBC, accompanied by alterations in the abundance of specific taxa, including increased abundance of genera such as *Veillonella* and *Lactobacillus* and decreased abundance of SCFA-producing taxa such as *Faecalibacterium* (Tang et al., 2018). Bidirectional Mendelian randomization analyses have further suggested a potential causal association between gut microbiota and PBC, with certain taxa, such as Coriobacteriales, possibly linked to an increased risk of disease (Zhou et al., 2024). Notably, an experimental study published in 2025 showed that transplantation of fecal microbiota from patients with PBC into mice could induce or aggravate liver phenotypes resembling PBC, thereby supporting a pathogenic role of gut microbiota in disease development (Jiang et al., 2024). However, no clinical trial results of fecal microbiota transplantation (FMT) in patients with PBC have yet been reported, and this area warrants further investigation.

Although multiple studies have identified gut microbiota alterations in PBC, the current body of evidence remains limited in several respects. Most available studies are cross-sectional and involve relatively small sample sizes. In addition, substantial differences in sequencing platforms, sample types, and analytical pipelines contribute to considerable heterogeneity across studies. Furthermore, the existing literature has been dominated by narrative reviews that qualitatively summarize current findings, while systematic quantitative synthesis remains lacking (Nie et al., 2025; Tang and You, 2025). A systematic review and meta-analysis published in 2025 applied Mendelian randomization to investigate the overall effects of gut microbiota on liver diseases, including PBC, but did not provide a comprehensive quantitative synthesis of PBC-associated diversity indices or multilevel taxonomic alterations (Xu et al., 2025).

However, several specific questions remain unresolved. First, it is unclear whether the reduction in gut microbial alpha diversity reported in individual studies is consistent across different cohorts, sequencing methods, and analytical settings. Second, beta-diversity findings have not been systematically compared across studies using different ecological distance metrics and reporting formats. Third, recurrent taxonomic signatures of PBC across different microbial levels remain incompletely summarized. Fourth, the extent to which age, geographic region, and sequencing platform contribute to between-study heterogeneity has not been quantitatively evaluated. Therefore, the present systematic review and meta-analysis aimed to quantitatively synthesize alpha-diversity indices, qualitatively summarize beta-diversity patterns, characterize recurrent taxonomic alterations across multiple taxonomic levels, and explore potential sources of heterogeneity through subgroup and meta-regression analyses. By doing so, this study provides a consolidated evidence base for future functional, longitudinal, and clinically oriented microbiome studies in PBC.

## 2 Materials and methods

This study was designed in accordance with the methodological recommendations of the Cochrane Collaboration (Higgins et al., 2019). The protocol was prospectively registered in the International Prospective Register of Systematic Reviews (PROSPERO) under registration number CRD420261344794 and is available at: https://www.crd.york.ac.uk/PROSPERO/view/CRD420261344794. The study was reported in accordance with the Preferred Reporting Items for Systematic Reviews and Meta-Analyses (PRISMA) 2020 statement (Page et al., 2021).

### 2.1 Search strategy

A systematic and structured literature search strategy was developed to identify studies investigating gut microbiota alterations in patients with PBC. The detailed search strategy and Boolean logic combinations are presented in Supplementary Table S1. PubMed, Web of Science, Embase, and the Cochrane Library were systematically searched from database inception to January 23, 2026 (the final search date). The search strategy combined controlled vocabulary terms (e.g., MeSH and Emtree) with free-text terms. The main search terms included “Primary Biliary Cholangitis,” “PBC,” “gut microbiota,” “gut microbiome,” “intestinal microbiota,” and “intestinal microflora,” and the search fields included titles, abstracts, and keywords. These terms were combined using Boolean operators (“AND” and “OR”). To minimize the risk of missing relevant studies, the reference lists of all included articles were also manually screened. The literature search was conducted independently by two authors (Ren and Zhao), and any disagreements were resolved through discussion.

### 2.2 Study screening

All retrieved records were imported into EndNote software for duplicate removal. Duplicate records that could not be identified automatically were further checked and removed manually by the investigators. Two reviewers (Ren and Zhao) independently screened the studies to determine their eligibility for inclusion. In the first stage, titles and abstracts were screened independently according to the predefined inclusion and exclusion criteria. In the second stage, the full texts of potentially relevant studies were retrieved and assessed in detail. Any disagreements between the two reviewers were resolved through discussion or consultation with a third reviewer (Xiong). The results of the screening process, including the number of included studies and the reasons for exclusion, were documented. The study selection process was reported in accordance with the PRISMA 2020 statement and is presented as a flow diagram showing the identification, screening, exclusion, and inclusion of studies at each stage.

### 2.3 Inclusion and exclusion criteria

*Inclusion criteria*: Studies were included if they (1) compared gut microbiota characteristics between patients with PBC and healthy controls; (2) were designed as prospective or retrospective cohort studies, or case–control studies; (3) reported data on gut microbiota composition and/or diversity in patients with PBC; and (4) had no language restrictions.

*Exclusion criteria*: Studies were excluded if they (1) were based on non-intestinal samples, such as blood, oral samples, or tissue specimens; (2) were case reports, reviews, comments, conference abstracts, or non-peer-reviewed publications; (3) did not include a healthy control group; (4) were duplicate publications or secondary analyses without original data; or (5) lacked quantitative data or reported only qualitative results.

### 2.4 Data extraction

Two reviewers (Ren and Zhao) independently extracted data from each included study and cross-checked the results to ensure consistency. Any discrepancies were resolved through discussion. All extracted information was recorded in a standardized data collection form. The extracted data included study characteristics (author, publication year, country, study design, sample size, sequencing method, sequencing platform, and sample type), population characteristics (e.g., age), and outcome measures (alpha diversity, beta diversity, and taxonomic findings). For results presented only in graphical form, the relevant data were extracted using WebPlotDigitizer 5.2.

### 2.5 Quality assessment

Two reviewers (Ren and Zhao) independently assessed the methodological quality of the included studies using the Newcastle–Ottawa Scale (NOS) (Stang, 2010), which is widely applied for the evaluation of non-randomized studies in systematic reviews and meta-analyses. Any disagreements were resolved by discussion or, if necessary, by consultation with a third reviewer (Xiong). Studies were categorized as high quality (NOS score ≥7), moderate quality (NOS score 5–6), or low quality (NOS score ≤4). The quality assessment was used to appraise the reliability of the included evidence and to explore potential sources of bias.

### 2.6 Statistical analysis

Meta-analysis and related figures were performed using Stata version 19.0, while heatmaps illustrating gut microbiota alterations across different taxonomic levels were generated using R version 4.5.2.

For continuous outcomes, including alpha-diversity indices such as the Shannon index, Simpson index, ACE index, Chao1 index, and operational taxonomic units (observed OTUs), pooled analyses were conducted using standardized mean differences (SMDs) with 95% confidence intervals (CIs), and Hedges’ g was applied to correct for small-sample bias. For studies reporting data as medians and interquartile ranges, values were converted into means and standard deviations according to the underlying data distribution. For approximately normally distributed data, the methods proposed by Wan et al. (2014) and Luo et al. (2018) were used, whereas for skewed data, the methods described by McGrath et al. (2020) and Cai et al. (2021) were applied. These converted values were treated as approximate estimates for quantitative synthesis, and the potential uncertainty introduced by these transformations was considered when interpreting the pooled results. To ensure statistical robustness, quantitative synthesis was performed only for outcomes reported in at least two independent studies.

Between-study heterogeneity was assessed using Cochran’s Q test and quantified with the I^2^ statistic (Irwig et al., 1998). Effect sizes were pooled using either a fixed-effect (common-effect) model or a random-effects model. When I^2^ ≤ 50%, indicating low heterogeneity, results from the fixed-effect model were reported; otherwise, results from the random-effects model were used. To further explore potential sources of heterogeneity, subgroup analyses and univariable random-effects meta-regression analyses were conducted. Prespecified subgroup factors included sequencing method, sequencing platform, and geographic region, while meta-regression covariates included region, mean age, and sequencing platform. When a covariate reached statistical significance in meta-regression (*p* < 0.05), the subgroup results were further examined to interpret its potential contribution to heterogeneity.

Beta diversity was qualitatively extracted to determine whether gut microbial communities in patients with PBC clustered differently from those in healthy controls. Because beta-diversity outcomes were reported using heterogeneous distance metrics, including unweighted UniFrac, weighted UniFrac, and Aitchison distance, and different reporting formats, including principal coordinates analysis (PCoA) plots, permutational multivariate analysis of variance (PERMANOVA) statistics, and descriptive statements of between-group separation, quantitative synthesis was not performed.

To characterize gut microbiota alterations in patients with PBC across the phylum, class, order, family, genus, and species levels, heatmaps were generated to visualize taxonomic changes. Only taxonomic alterations reported in at least two independent studies were summarized in order to improve consistency and interpretability.

For observed richness-related measures, including OTUs and observed features, these indicators were combined into a single richness category because both reflect the number of observed taxonomic units within a sample. However, given that their definitions and calculation methods are not entirely identical, the corresponding results should be interpreted with caution.

## 3 Results

### 3.1 Literature search

**Figure 1:**
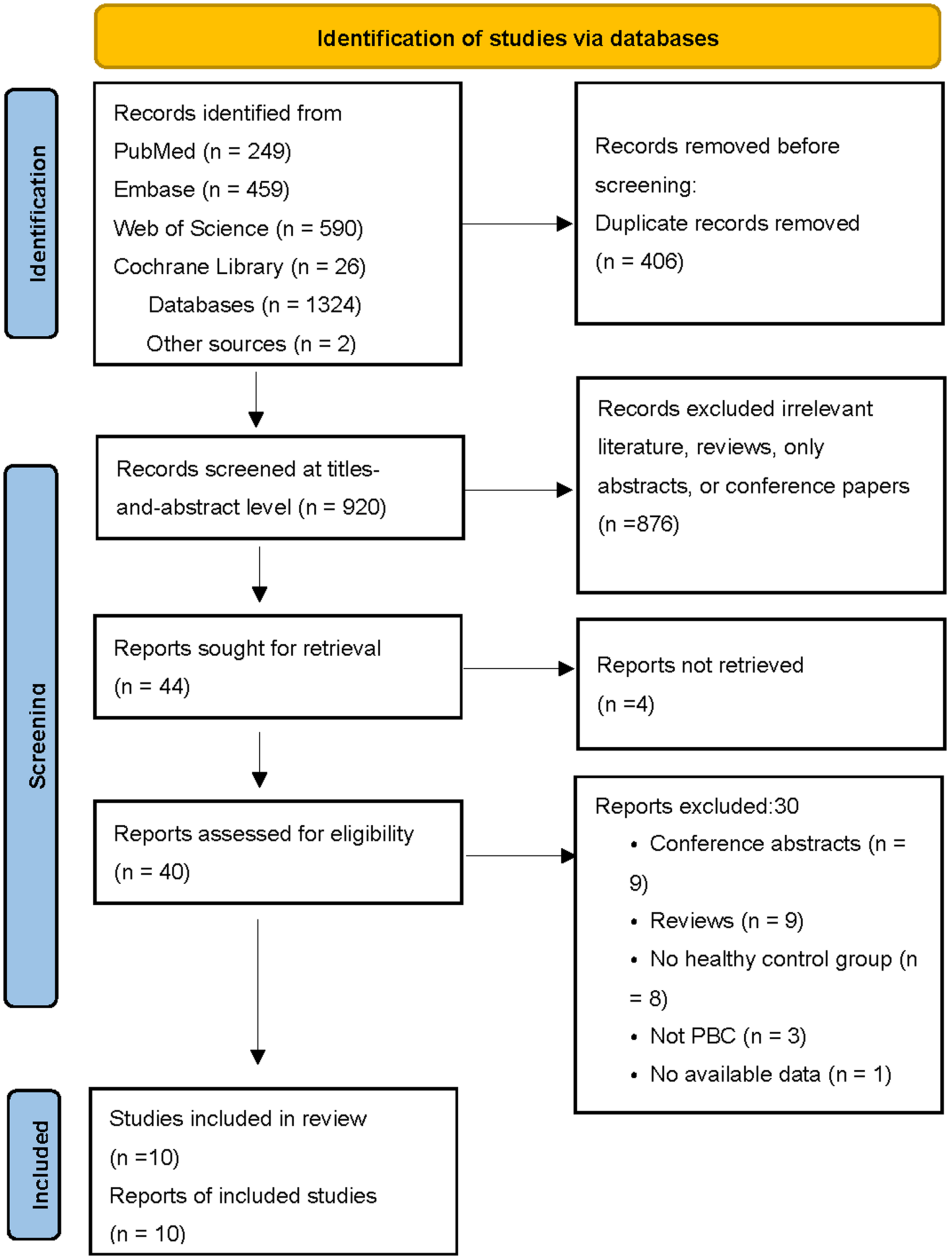
PRISMA flowchart of the study selection process.

A total of 1,326 records were retrieved through database searching. After duplicate removal using EndNote, supplemented by manual checking, 406 duplicate records were excluded, and 920 records remained for title and abstract screening. During the initial screening stage, 876 articles were excluded because they were irrelevant, review articles, conference abstracts, or abstract-only publications. Finally, 10 studies were included in both the qualitative and quantitative syntheses (Lv et al., 2016; Tang et al., 2018; Furukawa et al., 2020; Kitahata et al., 2021; Wang et al., 2021; Ding et al., 2022; Zhou et al., 2023; Wang Q. et al., 2024; Liu Q. Y. et al., 2025; Zang et al., 2025). The detailed study selection process is presented in Figure 1.

### 3.2 Study characteristics

A total of 10 studies investigating gut microbiota characteristics in patients with PBC were included in this review. These studies were published between 2016 and 2025 and were conducted mainly in China (8 studies) (Lv et al., 2016; Tang et al., 2018; Wang et al., 2021; Ding et al., 2022; Zhou et al., 2023; Liu Q. Y. et al., 2025; Zang et al., 2025; Wang Q. et al., 2024) and Japan (2 studies) (Furukawa et al., 2020; Kitahata et al., 2021). All included studies were based on independent populations with no overlap in participants, comprising a total of 1,057 individuals, including 607 patients with PBC and 450 healthy controls.

Regarding study design, all included studies were observational in nature. With the exception of Liu Q. Y. et al. (2025), which was designed as a prospective cohort study, the remaining studies were primarily case–control studies (Lv et al., 2016; Furukawa et al., 2020; Kitahata et al., 2021; Wang et al., 2021; Ding et al., 2022; Zhou et al., 2023; Wang Q. et al., 2024; Zang et al., 2025). Notably, the study by Tang et al. (2018) incorporated both a case–control component and a prospective follow-up design. No randomized controlled trials were identified.

The study populations mainly consisted of middle-aged and older adults. In most studies, the mean age of patients with PBC ranged from 46 to 66 years, whereas one study did not explicitly report age information (Liu Q. Y. et al., 2025). No studies involving children or infants were identified. Sample sizes varied substantially across studies, ranging from 9 to 132 participants in the PBC groups and from 8 to 131 participants in the control groups.

In terms of sequencing methodology, most studies (9/10) used 16S rRNA gene sequencing, with the V3–V4 region being the most commonly targeted region (Lv et al., 2016; Tang et al., 2018; Kitahata et al., 2021; Wang et al., 2021; Zhou et al., 2023; Wang Q. et al., 2024; Zang et al., 2025). Furukawa et al. (2020) used sequencing of the V1–V2 region, whereas Ding et al. (2022) did not clearly report the amplified region. Only Liu Q. Y. et al. (2025) applied shotgun metagenomic sequencing. The sequencing platforms were predominantly from the Illumina series, including MiSeq, NovaSeq, and HiSeq 2,500/PE150, with MiSeq being the most frequently used platform. Fecal samples were the predominant sample type, although one study used ileal mucosal samples (Kitahata et al., 2021). Given the potential ecological and compositional differences between mucosa-associated microbiota and fecal microbiota, this may represent a potential source of between-study heterogeneity.

With regard to alpha-diversity reporting, although all studies assessed gut microbial diversity, the specific indices varied across studies. The Shannon index was the most frequently reported metric, with data available from 7 studies (Lv et al., 2016; Furukawa et al., 2020; Wang et al., 2021; Ding et al., 2022; Wang Q. et al., 2024; Liu Q. Y. et al., 2025; Zang et al., 2025). The Chao1 index was reported in 6 studies (Lv et al., 2016; Kitahata et al., 2021; Wang et al., 2021; Ding et al., 2022; Zhou et al., 2023; Wang Q. et al., 2024), the ACE index in 3 studies (Wang et al., 2021; Zhou et al., 2023; Wang Q. et al., 2024), OTUs in 3 studies (Tang et al., 2018; Furukawa et al., 2020; Wang et al., 2021), and the Simpson index in 2 studies (Wang et al., 2021; Wang Q. et al., 2024). In addition, several studies reported other diversity measures, including PD_whole_tree (Furukawa et al., 2020), observed features (Zhou et al., 2023), and the Sobs and Coverage indices (Wang Q. et al., 2024). All 10 included studies had NOS scores of at least 7, indicating generally high methodological quality. The diversity data were presented either numerically or graphically and were therefore eligible for qualitative synthesis. Detailed characteristics of the included studies are summarized in Table 1.

**Table 1:** Characteristics of all the studies included in the meta-analysis.

Author, Year	Area	Study design	age (SD/range)	Case (PBC/HC)	Sequencing method	Sequencing Platform	Sample type	Alpha diversity index	Quality Score
Lv et al. (2016)	China	Case–control study	50.08 ± 1.30	42/30	16S rRNA gene sequencing (V3-V4 region)	Illumina MiSeq	Fecal samples	Shannon index, Chao 1	8
Zang et al. (2025)	China	Case–control study	PBC:46.24 ± 7.74 HC:44.85 ± 8.22	38/20	16S rRNA gene sequencing (V3-V4 region)	Illumina NovaSeq	Fecal samples	Shannon index	9
Furukawa et al. (2020)	Japan	Case–control study	PBC:66.0 ± 8.3 HC:60.5 ± 8.1	76/23	16S rRNA gene sequencing (V1-V2 region)	Illumina MiSeq	Fecal samples	OTUs, PD_whole_tree, Shannon index	8
Tang et al. (2018)	China	Case–control study and prospective study	PBC: 52 (22–78) HC: 47.5 (25–65)	60/80	16S rRNA gene sequencing (V3-V4 region)	Illumina MiSeq	Fecal samples	OTUs, Shannon index	9
Zhou et al. (2023)	China	Case–control study	PBC: 55 (43–77) HC: 57 (24–77)	25/25	16S rRNA gene sequencing (V3-V4 region)	Illumina NovaSeq 6,000	Fecal samples	ACE, Chao 1, Observed features	9
Liu M. et al. (2025)	China	Prospective cohort study	Not specified	132/131	Shotgun metagenomic sequencing	Illumina NovaSeq 6,000	Fecal samples	Shannon index	9
Kitahata et al. (2021)	Japan	Case–control study	PBC: 66 (58–72) HC: 69 (55–73)	34/21	16S rRNA gene sequencing (V3-V4 region)	Illumina MiSeq	Ileal mucosal	Chao 1, Shannon index	9
Wang Q. et al. (2024)	China	Case–control study	PBC:53.77 ± 10.19 HC: 54.69 ± 8.22	105/26	16S rRNA gene sequencing (V3-V4 region)	Illumina HiSeq 2,500	Fecal samples	Chao 1, ACE, Sobs, Shannon index, Simpson, Coverage	8
Wang et al. (2021)	China	Case–control study	PBC: 53.14 ± 2.84 HC: 53.21 ± 2.35	9/8	16S rRNA gene sequencing (V3-V4 region)	Illumina HiSeq 2,500	Fecal samples	ACE, Chao 1, Simpson, Shannon index	7
Ding et al. (2022)	China	Case–control study	PBC: 46.73 ± 4.12 HC: 47.14 ± 3.98	86/86	16S rRNA gene sequencing	Illumina HiSeq PE150	Fecal samples	Chao 1, Shannon index	8

### 3.3 Changes in alpha-diversity in PBC

**Figure 2:**
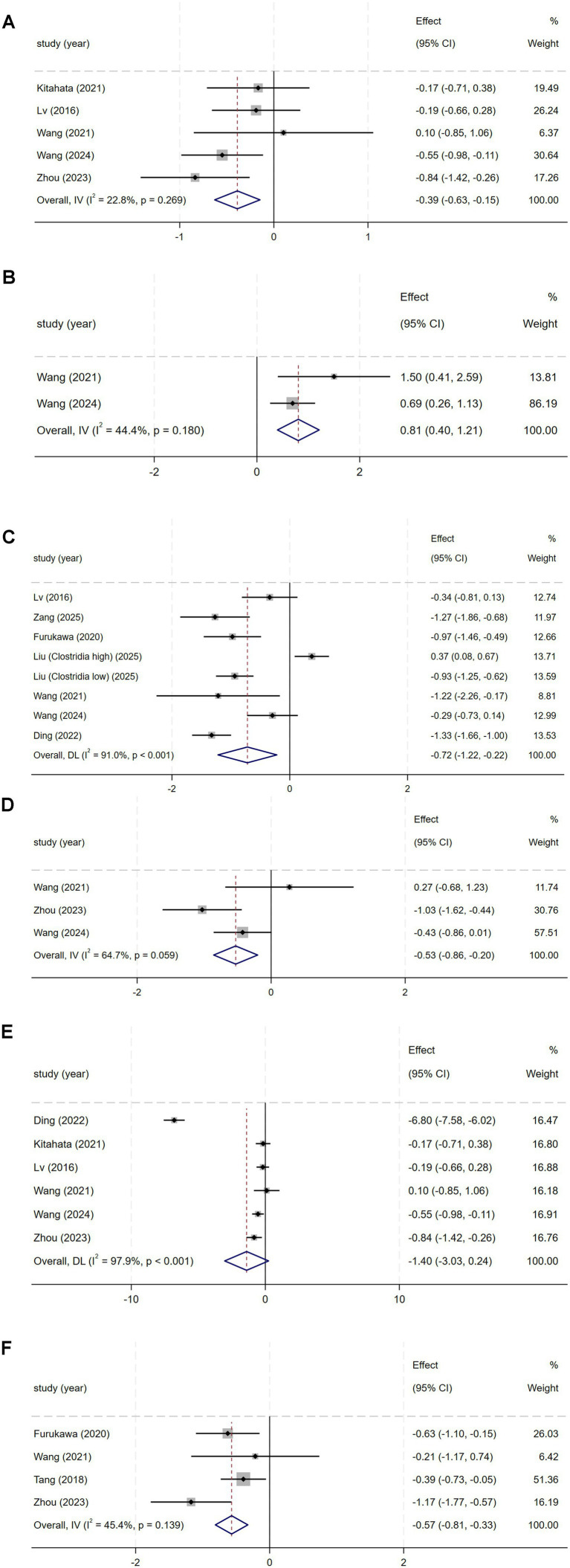
Forest plots of changes in α-diversity indices of the gut microbiota in patients with PBC. **(A)** Shannon index. **(B)** Chao1 index. **(C)** OTUs. **(D)** Simpson index. **(E)** ACE index. **(F)** Chao1 index after exclusion of the study by Ding et al. (2022). Weights are from random-effects model.

In patients with PBC, the reported alpha-diversity indices mainly included the Shannon index, Chao1 index, OTUs, Simpson index, and ACE index (Figure 2). The analytical methods and detailed results of each study are summarized in Supplementary Table S2. It should be noted that Liu Q. Y. et al. (2025) further divided patients with PBC into Clostridia^+^ and Clostridia^−^ subgroups and compared each subgroup separately with the healthy control group. Because these two subgroups were derived from the same study cohort and shared a common control group, this data structure may introduce a risk of non-independence of effect sizes in the quantitative synthesis, and the corresponding results should therefore be interpreted with caution.

**Figure 3:**
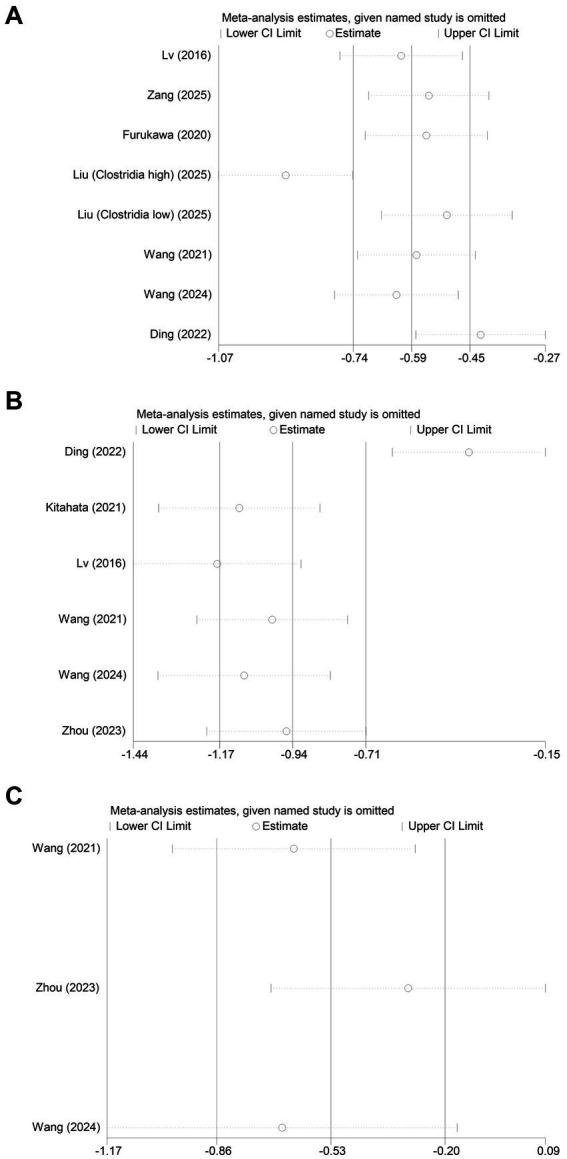
Leave-one-out sensitivity analysis of the pooled effect sizes for gut microbiota diversity in patients with PBC. **(A)** Shannon index. **(B)** Chao1 index. **(C)** ACE index.

**Figure 4:**
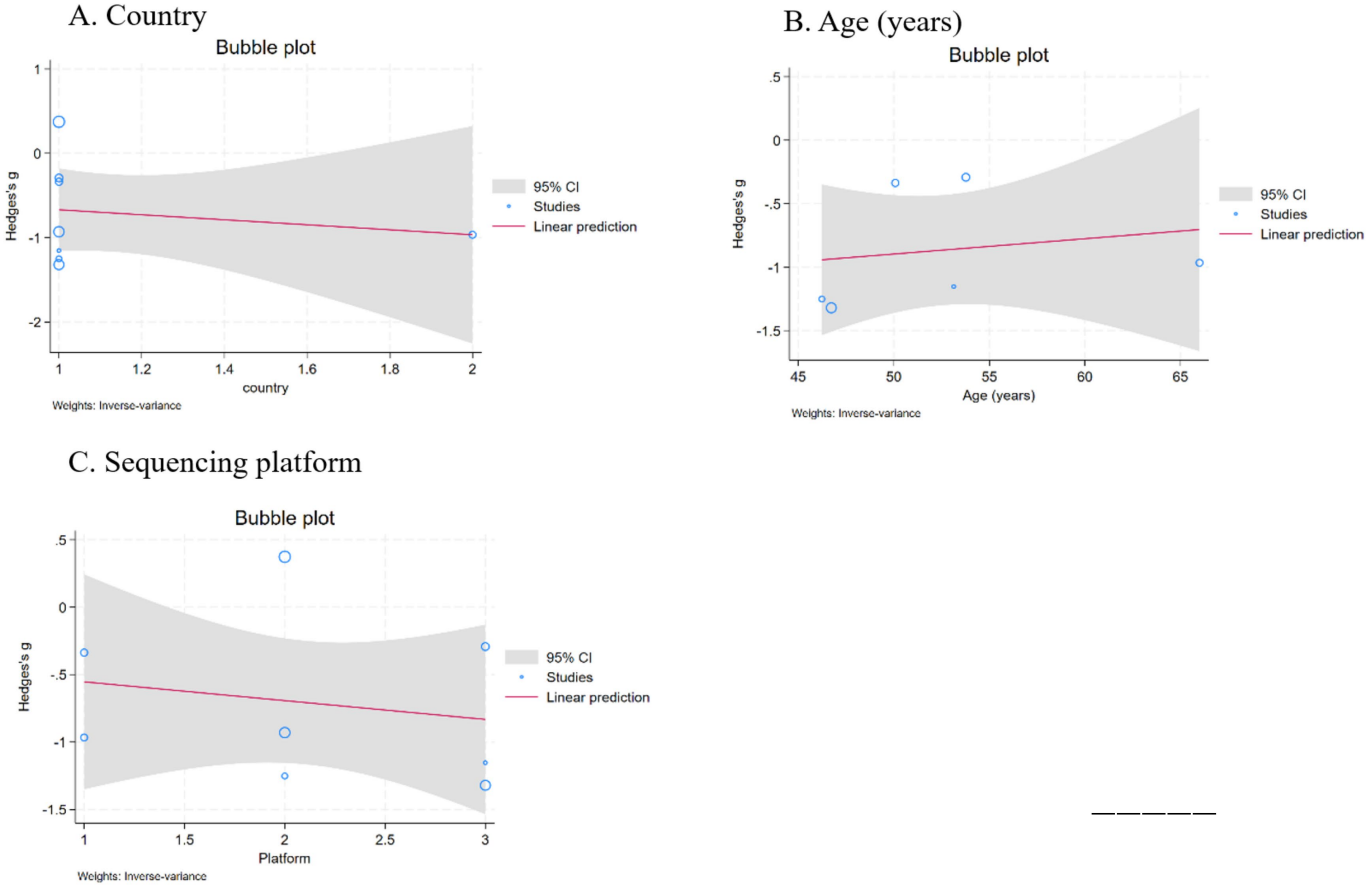
Meta-regression analysis of the Shannon diversity index in the gut microbiota of patients with PBC **(A)** Country, **(B)** Age (years), **(C)** Sequencing platform.

A total of 7 studies reported the Shannon index (Figure 2A). Random-effects meta-analysis showed that the Shannon index of the gut microbiota was significantly lower in patients with PBC than in healthy controls (SMD = −0.72, 95% CI: −1.22 to −0.22, *p* < 0.001), although substantial heterogeneity was observed across studies (I^2^ = 91.0%, p < 0.001). To investigate the potential sources of heterogeneity, we first conducted a sensitivity analysis (Figure 3A). The pooled effect sizes ranged from −1.07 to −0.27, and none of the corresponding 95% CIs crossed the null value, indicating that the finding of a significantly reduced Shannon index in PBC was robust. We then performed subgroup analyses (Table 2) and meta-regression (Figure 4). Subgroup analyses stratified by sequencing platform, geographic region, and sequencing method did not explain the high between-study heterogeneity. In most subgroups, the conclusion that the Shannon index was significantly reduced in patients with PBC remained unchanged, further supporting the robustness of the main finding. Meta-regression also showed that country, age, and sequencing platform were not significant contributors to the observed heterogeneity, suggesting that other unmeasured clinical or technical factors may be involved.

**Table 2:** Subgroup analysis of Shannon diversity in PBC vs. healthy gut microbiomes.

Subgroup	*n*	Mean/Effect	95% CI	Heterogeneity $I^{2}$	*p* (subgroups)
Sequencing platform					0.843
MiSeq	2	−0.65	−1.27, −0.03	70.1%	
NovaSeq	3	−0.59	−1.62, 0.44	95.7%	
HiSeq	3	−0.92	−1.71, −0.13	85.8%	
Area					0.443
China	7	−0.68	−1.24, −0.13	92.0%	
Japan	1	−0.97	−1.46, −0.49	0.0%	
Sequencing method					0.386
16S rRNA gene sequencing	6	−0.88	−1.30, −0.45	76.5%	
Shotgun metagenomic sequencing	2	−0.28	−1.56, 1.00	97.2%	
Overall	8	−0.72	−1.22, −0.22	91.0%	P < 0.001

A total of 6 studies reported the Chao1 index (Figure 2B). Random-effects meta-analysis indicated no statistically significant difference in the Chao1 index between patients with PBC and healthy controls (SMD = −1.40, 95% CI: −3.03 to 0.24, *p* > 0.05), with substantial heterogeneity across studies (I^2^ = 97.9%, *p* < 0.001). Sensitivity analysis identified Ding et al. (2022) as a potential outlier (Figure 3B). After exclusion of this study, fixed-effect meta-analysis of the remaining 5 studies showed a statistically significant reduction in the Chao1 index in patients with PBC (Figure 2F; SMD = −0.38, 95% CI: −0.63 to −0.15, p < 0.001), and heterogeneity was markedly reduced (I^2^ = 22.6%, *p* = 0.269). Notably, Kitahata et al. (2021) was the only study using ileal mucosal samples rather than fecal samples. However, leave-one-out sensitivity analysis showed that excluding this study did not materially change the overall direction of the pooled Chao1 estimate, suggesting that the Chao1 finding was not primarily driven by this mucosal-sample study.

A total of 4 studies reported observed richness-related measures, including 3 studies reporting OTUs and 1 study reporting observed features. Because both metrics reflect microbial richness, they were combined into a single OTU/richness category for pooled analysis (Figure 2C). Fixed-effect meta-analysis showed that OTUs were significantly lower in patients with PBC than in healthy controls (SMD = −0.57, 95% CI: −0.81 to −0.33, p < 0.001), with low between-study heterogeneity (I^2^ = 45.4%, *p* = 0.139).

Only 2 studies reported the Simpson index (Figure 2D). Fixed-effect meta-analysis demonstrated that the Simpson index was significantly higher in patients with PBC than in healthy controls (SMD = 0.81, 95% CI: 0.40 to 1.21, p < 0.001), with low heterogeneity (I^2^ = 44.4%, *p* = 0.180).

A total of 3 studies reported the ACE index (Figure 2E). Random-effects meta-analysis showed no statistically significant difference in the ACE index between patients with PBC and healthy controls (SMD = −0.44, 95% CI: −1.09 to 0.13, p > 0.05), while moderate heterogeneity was present (I^2^ = 64.7%, *p* = 0.059). To further investigate the potential source of heterogeneity, a sensitivity analysis was performed (Figure 3C). The sensitivity analysis for the ACE index showed that the overall direction of the pooled effect remained generally consistent; however, the 95% CI crossed zero in some leave-one-out analyses, indicating limited stability of this finding.

Taken together, these results indicate that the gut microbiota of patients with PBC exhibits marked alterations in alpha diversity. Multiple indices, including the Shannon index, Chao1 index, and OTUs, suggested reduced diversity and/or richness, whereas the Simpson index was significantly increased and the ACE index showed no statistically significant difference. Overall, the gut microbial ecosystem in PBC appears to be characterized by reduced diversity and decreased species richness, reflecting substantial microbial dysbiosis. Because some alpha-diversity data were converted from medians and interquartile ranges or extracted from graphical presentations, the pooled estimates should be interpreted with appropriate caution.

### 3.4 Summary of beta-diversity outcomes

A total of 8 studies were included in the analysis of beta diversity in the gut microbiota of patients with PBC. The analytical methods and detailed results of each study are summarized in Supplementary Table S3. The included studies mainly assessed beta diversity using unweighted UniFrac, weighted UniFrac, and Aitchison distance, combined with PCoA and PERMANOVA, to compare differences in gut microbial community composition between patients with PBC and healthy controls.

Most studies reported significant differences in gut microbial community structure between patients with PBC and healthy controls. Specifically, Furukawa et al. (2020), Zhou et al. (2023), Wang Q. et al. (2024), and Zang et al. (2025) identified statistically significant differences in gut microbiota composition between the two groups based on weighted UniFrac analysis. Tang et al. (2018) obtained a consistent result using unweighted UniFrac, while Liu Q. Y. et al. (2025) also observed a marked difference in microbial community composition between patients with PBC and controls using Aitchison distance. These findings suggest that the development of PBC is accompanied by alterations in gut microbial community structure.

At the same time, some heterogeneity in beta-diversity findings was observed across studies. Lv et al. (2016) and Kitahata et al. (2021) used unweighted UniFrac analysis but did not detect significant differences in microbial community structure between the two groups. In addition, Wang et al. (2021) and Ding et al. (2022) did not report any beta-diversity-related data. The discrepancies between results obtained using different analytical approaches, particularly unweighted UniFrac versus weighted UniFrac, further highlight the methodological complexity of beta-diversity assessment.

Overall, although some between-study heterogeneity was present, the available evidence suggests that beta diversity is altered in patients with PBC, indicating structural dysbiosis of the gut microbial ecosystem in this disease.

### 3.5 The changes in the bacterial community at the taxonomic level

Among the 10 studies investigating gut microbiota in patients with PBC, 9 studies reported significant differences between patients and controls at the phylum, family, genus, or species levels. Given the potential for false-positive findings, we summarized only those taxa at the genus level that were reported in at least two independent studies, and classified the findings as Increased, Inconsistent, Decreased, or NS (no significant difference). Taxa at other taxonomic levels that were not specifically listed are presented in Supplementary Figure S1.

#### 3.5.1 Changes in taxonomic composition at the phylum level associated with PBC

**Figure 5:**
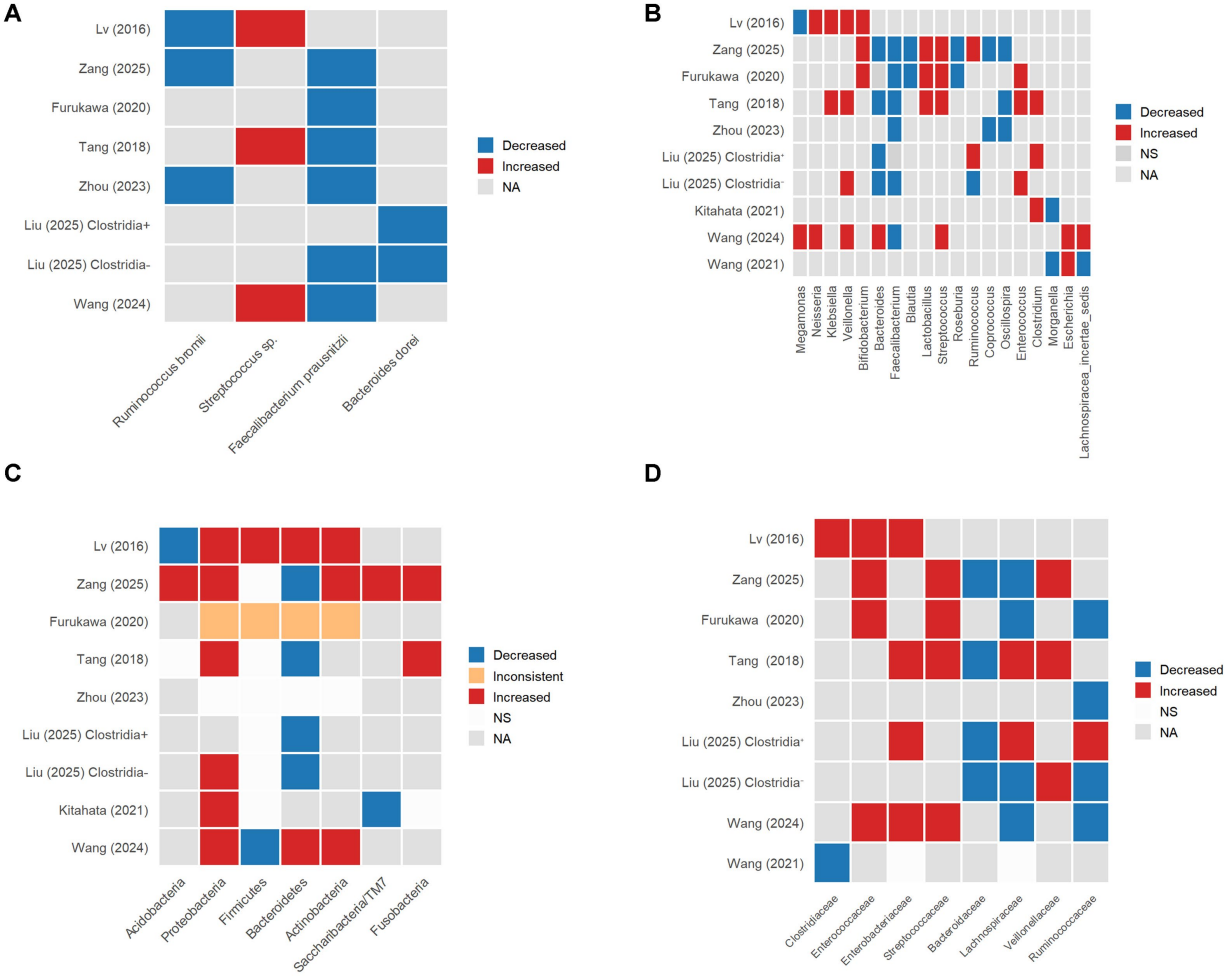
Changes in gut microbiota composition in PBC across multiple taxonomic levels: **(A)** Phylum level, **(B)** Family level, **(C)** Genus level, and **(D)** Species level. NS, no significant difference; NA, not reported.

Figure 5A summarizes the phylum-level taxonomic alterations reported across studies. At the phylum level, the gut microbiota of patients with PBC was characterized by relative enrichment of Proteobacteria, Actinobacteria, and Fusobacteria, whereas Bacteroidetes tended to be reduced in some studies. Changes in Acidobacteria, Firmicutes, and Saccharibacteria/TM7 were inconsistent across studies.

#### 3.5.2 Changes in taxonomic composition at the family level associated with PBC

Figure 5B presents the family-level taxonomic changes reported in patients with PBC. At the family level, Enterococcaceae, Enterobacteriaceae, Streptococcaceae, and *Veillonellaceae* were generally enriched in patients with PBC, whereas Bacteroidaceae and Ruminococcaceae were predominantly decreased. In contrast, findings for Lachnospiraceae and Clostridiaceae were inconsistent across studies.

#### 3.5.3 Changes in taxonomic composition at the genus level associated with PBC

Figure 5C illustrates the genus-level taxonomic changes associated with PBC. At the genus level, *Neisseria*, *Klebsiella*, *Veillonella*, *Bifidobacterium*, *Lactobacillus*, *Streptococcus*, *Enterococcus*, *Clostridium*, and *Escherichia* were relatively enriched in patients with PBC. In contrast, *Bacteroides*, *Faecalibacterium*, *Blautia*, *Roseburia*, *Coprococcus*, *Oscillospira*, and *Morganella* were generally reported to be reduced in patients with PBC. The direction of change for genera such as *Megamonas* and *Ruminococcus* was inconsistent across studies. However, the direction and statistical significance of some taxonomic changes varied across individual studies; therefore, these genus-level findings should be interpreted as recurrent trends rather than uniform changes across all cohorts.

#### 3.5.4 Changes in taxonomic composition at the species level associated with PBC

Figure 5D shows the species-level taxonomic alterations reported in patients with PBC. At the species level, *Streptococcus* sp. was enriched in patients with PBC, whereas *Ruminococcus bromii*, *Faecalibacterium prausnitzii*, and *Bacteroides dorei* were decreased.

### 3.6 Risk of bias assessment

A total of 10 studies were included in the meta-analysis, and the number of studies contributing to each outcome did not exceed 10. Owing to the limited number of included studies for each indicator, funnel plots were not generated to assess publication bias. The corresponding sensitivity analyses and meta-regression analyses have been described in detail in the preceding sections.

## 4 Discussion

In this meta-analysis including 10 studies, we systematically evaluated the characteristics of gut microbiota alterations in patients with PBC and their potential association with the disease. Three major findings emerged from the present study. First, alpha diversity of the gut microbiota showed an overall decreasing trend in patients with PBC. Specifically, the Shannon index was significantly reduced (SMD = −0.72, 95% CI: −1.22 to −0.22), and although the Chao1 index did not reach statistical significance in the overall analysis, it showed a significant reduction after exclusion of an outlier study in sensitivity analysis (SMD = −0.38, 95% CI: −0.63 to −0.15). In addition, OTUs were significantly decreased (SMD = −0.57, 95% CI: −0.81 to −0.33, *p* < 0.001), whereas the Simpson index was significantly increased (SMD = 0.81, 95% CI: 0.40 to 1.21, p < 0.001), suggesting reduced microbial diversity accompanied by an increased relative abundance of dominant taxa. Second, most studies assessing beta diversity using UniFrac or Aitchison distance demonstrated clear differences in gut microbial community structure between patients with PBC and healthy controls, indicating an overall shift in microbial ecological composition associated with PBC. Third, at the taxonomic level, the gut microbiota of patients with PBC exhibited a pattern of systematic dysbiosis. At the genus level, potentially pathogenic taxa such as *Klebsiella*, *Veillonella*, *Streptococcus*, and *Escherichia* were enriched, whereas short-chain fatty acid (SCFA)-producing taxa, including *Faecalibacterium*, *Bacteroides*, and *Roseburia*, were depleted. Collectively, these findings suggest that the gut microbial ecosystem in PBC is characterized by an imbalance marked by enrichment of potential pathogens and depletion of beneficial anti-inflammatory commensals. The main contribution of this study is the integration of scattered PBC microbiome findings into a structured evidence framework covering alpha diversity, beta diversity, taxonomic alterations, and heterogeneity exploration.

### 4.1 Changes in gut microbiota diversity indices

Several previous reviews have discussed the alterations and potential role of the gut microbiota in PBC (Zhang et al., 2023; Tanaka et al., 2024; Nie et al., 2025; Tang and You, 2025; Zhao and Lian, 2025). However, to the best of our knowledge, no previous study has systematically synthesized and quantitatively evaluated both alpha and beta diversity of the gut microbiota in patients with PBC through a systematic review and meta-analysis. Alpha diversity is a key ecological metric that reflects species richness and evenness within a microbial community and is widely used to assess gut microbial diversity (Gevers et al., 2012; Hagerty et al., 2020). In the present study, both the Shannon index and OTU/observed richness-related measures were lower in patients with PBC than in healthy controls. Although the Chao1 index did not reach statistical significance in the primary analysis, it showed a significant decrease after exclusion of an outlier study.

These indices reflect different aspects of gut microbial ecology. The Shannon index captures both species richness and evenness, whereas Chao1 and OTUs mainly reflect microbial richness (Chen et al., 2025). By contrast, the significantly increased Simpson index suggests reduced microbial diversity accompanied by a greater relative abundance of dominant taxa. This finding was consistent with the original studies, although only two studies contributed data for this index, which limits the reliability of the result (Wang et al., 2021; Wang Q. et al., 2024). The ACE index did not show a statistically significant difference, which may be related to its sensitivity to taxa with different abundance distributions, the limited number of included studies, and the relatively high between-study heterogeneity. Notably, the number of studies available for most alpha-diversity indices was small, particularly for ACE (three studies) and Simpson (two studies). In addition, substantial heterogeneity was observed for both the Shannon and ACE indices. Although subgroup analyses and meta-regression were further conducted, the sources of heterogeneity could not be fully explained. Previous meta-analyses of the gut microbiota have suggested that body mass index (BMI) may be an important source of heterogeneity; however, this factor could not be explored in the present study because BMI data were unavailable in several included studies (Wang et al., 2025). Therefore, BMI may have contributed to the unexplained heterogeneity observed here. These limitations should be considered when interpreting the findings of the present study.

### 4.2 Alterations in characteristic gut microbiota

The gut microbiota of patients with PBC undergoes marked alterations across multiple taxonomic levels, reflecting a complex pattern of microbial dysbiosis. Gut microbes may participate in the pathogenesis and progression of PBC through several mechanisms, most of which converge on regulation of the gut–liver axis (Zhang et al., 2023; Ichikawa et al., 2024). Overall, beneficial commensals tend to be depleted in PBC, whereas opportunistic pathogens are enriched.

At the genus level, our analysis identified substantial compositional changes in the gut microbiota of patients with PBC. The genera most commonly reported as depleted included *Bacteroides*, *Faecalibacterium*, *Blautia*, *Roseburia*, *Coprococcus*, *Oscillospira*, and *Morganella*, although the consistency and statistical significance of these changes varied among individual studies. Many of these taxa belong to the Lachnospiraceae and Ruminococcaceae families within the phylum Firmicutes and are important producers of SCFAs, including butyrate, propionate, and acetate. In contrast, enriched genera included *Neisseria*, *Klebsiella*, *Veillonella*, *Bifidobacterium*, *Lactobacillus*, *Streptococcus*, *Enterococcus*, *Clostridium*, and *Escherichia*. Some Gram-negative taxa, such as Klebsiella, Escherichia, and Neisseria, may be associated with lipopolysaccharide (LPS)- or lipooligosaccharide (LOS)-related pro-inflammatory signaling, whereas Bifidobacterium and Lactobacillus are generally considered probiotic-associated genera and should not be generalized as potentially pathogenic solely on the basis of increased abundance. It should be noted that the present taxonomic analysis was primarily conducted at the genus level, and functional inference based solely on taxonomic composition has inherent limitations, because species within the same genus may differ substantially in biological function. Therefore, these findings require further confirmation through functional validation or metabolite profiling.

Gut microbiota play a central role in bile acid metabolism, influencing the bile acid pool through bile salt hydrolase (BSH) activity and 7α-dehydroxylation, thereby affecting liver physiology (Guo et al., 2024; Zhang et al., 2025). *Clostridium* was enriched in patients with PBC. Certain members of this genus, particularly *Clostridium scindens*, possess highly efficient 7*α*-dehydroxylase activity and can convert primary bile acids into secondary bile acids. For example, obeticholic acid (OCA) can be 7α-dehydroxylated into lithocholic acid (LCA), a process that may represent a key mechanism underlying the enhanced hepatotoxicity of OCA (Zhang et al., 2025). LCA is a hydrophobic secondary bile acid whose accumulation in the liver is cytotoxic and may induce mitochondrial dysfunction and oxidative stress, thereby aggravating cholangiocyte injury (Fickert et al., 2006). In patients with PBC, cholestasis alters the intestinal bile acid milieu and may create a favorable niche for the expansion of certain *Clostridium* species (Zhang et al., 2025). Enrichment of these taxa may in turn promote abnormal accumulation of toxic secondary bile acids such as LCA, which may not only directly damage bile duct epithelial cells but also modulate the balance between T helper 17 (Th17) cells and regulatory T (Treg) cells, thereby exacerbating immune dysregulation (Hang et al., 2019). At the same time, because OCA is used therapeutically in PBC, the potential generation of LCA through microbial 7*α*-dehydroxylation may contribute to hepatotoxicity and thus limit its clinical application.

SCFAs, particularly butyrate, are fermentation products of dietary fiber generated by intestinal bacteria and are essential for maintaining intestinal barrier integrity, regulating immune function, and suppressing inflammation (Wang R. et al., 2024). In patients with PBC, several depleted genera, including *Faecalibacterium*, *Blautia*, *Roseburia*, *Coprococcus*, and *Oscillospira*, belong mainly to the Lachnospiraceae and Ruminococcaceae families and represent major SCFA-producing or SCFA-associated taxa (Dicks, 2024). These bacteria produce metabolites such as butyrate, propionate, and acetate, which play critical roles in preserving intestinal barrier function and immune homeostasis. Butyrate may be particularly important in alleviating PBC by inducing epigenetic and metabolic reprogramming of myeloid-derived suppressor cells (MDSCs), thereby modulating immune dysregulation (Wang R. et al., 2024). The reduction of these SCFA-producing bacteria in PBC may lower intestinal butyrate levels, weaken epithelial barrier function, and increase intestinal permeability (“leaky gut”). Barrier disruption may facilitate the translocation of bacteria and bacterial products, such as LPS, into the liver, thereby triggering inflammatory responses (Tang et al., 2018; Zhou et al., 2023). Reduced butyrate availability may also impair the maturation and function of immune cells, particularly Treg cells, thereby aggravating autoimmune inflammation in PBC (Hang et al., 2019; Wang R. et al., 2024).

LPS is a major component of the cell wall of Gram-negative bacteria and has potent immunostimulatory properties. Once it enters the circulation, LPS can activate the host immune system and promote inflammatory responses (Guo et al., 2024; Liu M. et al., 2025). In the present study, enriched genera in PBC included *Neisseria*, *Klebsiella*, *Streptococcus*, *Enterococcus*, and *Escherichia*. Among these, *Escherichia* and *Klebsiella* belong to Proteobacteria, are Gram-negative bacteria, and contain LPS in their outer membrane (Tume et al., 2025). Although *Streptococcus* and *Enterococcus* are Gram-positive bacteria, certain strains may also promote inflammation or bacterial translocation through alternative mechanisms. For example, lipoteichoic acid (LTA) from *Streptococcus mutans* can activate macrophages via TLR2 and induce the release of inflammatory mediators such as TNF-α and nitric oxide (Hong et al., 2014). In *Enterococcus faecalis*, LTA may act synergistically with SCFAs such as butyrate to enhance inflammasome activation and IL-1β release, underscoring the pro-inflammatory role of Gram-positive bacterial components (Park et al., 2023). *Neisseria*, as a Gram-negative genus, contains LOS, which functions as an endotoxin and activates pro-inflammatory signaling through Toll-like receptor 4 (TLR4) / myeloid differentiation factor 2 (MD-2) recognition (Liu et al., 2010). Because PBC is frequently accompanied by impaired intestinal barrier function and increased permeability, these enriched bacteria and their immunogenic products are more likely to translocate into the portal circulation and reach the liver. There, LPS may activate the TLR4 signaling pathway, inducing hepatocytes and immune cells to release pro-inflammatory cytokines such as IL-6 and TNF-α, thereby aggravating hepatic inflammation and bile duct injury (Li and Liu, 2025). Supporting this concept, a recent study in a mouse model of cholestatic liver disease showed that *Lactococcus garvieae* could worsen disease by increasing intestinal permeability and promoting bile acid reabsorption, indirectly supporting a mechanistic link between bacterial translocation and inflammation (Liu M. et al., 2025).

From the perspective of identifying potential mechanisms underlying autoimmune injury to the biliary system, further interpretation of the gut microbiota alterations identified in this meta-analysis is of practical significance. Unlike general mechanisms shared by many liver diseases, such as bile acid dysregulation, impaired intestinal barrier function, endotoxin-related inflammation, and immune dysregulation, antigenic mimicry may represent a more PBC-specific link between gut dysbiosis and autoimmune cholangitis. Previous studies have suggested that intestinal microbiota may contribute to autoimmune liver injury by regulating microbial antigen exposure, mucosal immune responses, and immune tolerance, while pathogen-derived antigens may trigger or perpetuate PBC-related autoimmunity through molecular mimicry with host mitochondrial autoantigens (Ma et al., 2015; Tanaka et al., 2019). In this context, the enrichment of Streptococcus observed in the present meta-analysis is noteworthy. Haruta et al. (2008) reported increased IgM reactivity against *Streptococcus intermedius* and its histone-like DNA-binding protein in patients with PBC, and immunoreactivity to this bacterial antigen was detected in biliary epithelial cells and inflammatory cells in portal areas, suggesting a possible role of *S. intermedius*-related antigens in bile duct injury through immune cross-reactivity. Although our genus-level analysis cannot determine whether *S. intermedius* was specifically enriched or whether antigenic mimicry directly occurred, these findings suggest that altered microbial antigenic exposure may represent a potential PBC-specific mechanism linking gut dysbiosis to autoimmune cholangitis. Future studies integrating shotgun metagenomics, microbial antigen profiling, epitope prediction, autoantibody mapping, and T-cell response assays are needed to clarify this possibility.

Tryptophan is an essential amino acid whose metabolites are closely involved in intestinal homeostasis and immune regulation. The gut microbiota plays an important role in tryptophan metabolism by generating indole and its derivatives, which influence barrier function and immune responses (Roager and Licht, 2018). Enriched genera such as *Bifidobacterium*, *Lactobacillus*, and *Clostridium* may directly produce indole-3-lactic acid, indole-3-aldehyde, or related metabolites, whereas *Bacteroides*, one of the major taxa associated with tryptophanase activity, showed a decreasing trend (Wang et al., 2019). Gut dysbiosis may therefore lead to abnormalities in tryptophan metabolism, including reduced production of beneficial indole compounds or increased generation of potentially harmful metabolites (Guo et al., 2024). Indole derivatives are generally important for maintaining intestinal barrier function and modulating immune responses. Altered tryptophan metabolism may thus further aggravate intestinal barrier dysfunction and immune dysregulation, thereby promoting PBC progression. Although *Bifidobacterium*, *Lactobacillus*, and some *Clostridium* species were enriched and may produce indole-related metabolites, the decrease in *Bacteroides* may result in insufficient overall activation of the aryl hydrocarbon receptor (AhR). Taken together, gut microbiota dysbiosis may weaken the protective effects of tryptophan metabolism on intestinal barrier integrity and immune homeostasis (Roager and Licht, 2018).

In patients with PBC, the abundance of specific gut microbial taxa has also been reported to correlate significantly with liver function parameters, serum cytokine levels, and urinary metabolites. Specifically, *Veillonella* has been positively associated with IL-18 and IL-16, *Klebsiella* abundance has been correlated with IL-2A and total bilirubin, *Neisseria* has shown a positive association with urinary indole-3-propionate ester, and *Bifidobacterium* has been positively associated with immunoglobulin A (IgA) levels (Lv et al., 2016; Tang et al., 2018; Zang et al., 2025). In addition, increased abundance of *Streptococcus* has been proposed as a potential biomarker of increased cirrhosis risk in disease severity assessment (Wang Q. et al., 2024). Notably, *Bifidobacterium* is generally considered a beneficial probiotic genus that exerts hepatoprotective effects by producing SCFAs such as acetate and butyrate, thereby reducing oxidative stress, enhancing immune homeostasis, preserving gut barrier integrity, and attenuating systemic inflammation (Lim and Shin, 2020; Hizo and Rampelotto, 2024). Interestingly, however, *Bifidobacterium* abundance was higher in the PBC group than in healthy controls. One possible explanation is that competitive depletion of other beneficial commensals may create ecological space for the expansion of *Bifidobacterium*. In addition, chronic cholestasis in PBC may suppress bile acid-sensitive taxa, including certain members of the Clostridiaceae, thereby indirectly favoring the enrichment of *Bifidobacterium*.

Recent international evidence further supports the potential clinical relevance of microbiota-related alterations in PBC. Martinez-Gili et al. (2023) analyzed bacterial and bile acid metabolic phenotypes in patients with PBC and showed that these profiles were associated with inadequate biochemical response to UDCA, suggesting that microbiota-related metabolic features may have clinical implications beyond East Asian populations. In addition, broader evidence from autoimmune liver diseases also supports the involvement of the gut microbiome in immune-mediated liver injury. Schneider et al. (2024) summarized that autoimmune liver diseases, including PBC, autoimmune hepatitis, and primary sclerosing cholangitis, are generally associated with reduced gut microbial diversity and altered abundance of specific bacterial taxa. However, microbial signatures may differ according to population background, diet, disease stage, treatment exposure, and sequencing methodology. Therefore, the microbial features identified in the present meta-analysis should be interpreted as current evidence-based PBC-associated patterns rather than universally validated biomarkers.

Overall, gut microbiota dysbiosis in PBC appears to be a highly complex process. Enrichment of bile acid-transforming bacteria such as Clostridial taxa may increase toxic secondary bile acids, directly injure cholangiocytes, and disrupt immune balance (Zhang et al., 2025). Reduction of SCFA-producing bacteria may weaken the intestinal barrier and impair immunoregulation, thereby amplifying inflammation (Wang R. et al., 2024). Enrichment of LPS-associated bacteria, together with barrier dysfunction, may facilitate translocation of microbial products into the liver and activate the TLR4 signaling pathway, leading to persistent inflammation (Liu M. et al., 2025). Altered microbial regulation of tryptophan metabolism may further impair barrier integrity and immune homeostasis. Taken together, these mechanisms are likely to interact and collectively contribute to the onset and progression of PBC.

### 4.3 Functional and clinical implications of gut microbiota alterations in PBC

Although the present study mainly synthesized gut microbiota alterations at the diversity and taxonomic levels, several included studies provided preliminary functional evidence linking microbial dysbiosis with metabolic and immune disturbances in PBC. Lv et al. (2016) integrated gut microbiota profiling with metabolomic and immune-factor analyses and reported associations between altered microbial taxa, host metabolites, and immune parameters, suggesting that gut dysbiosis in PBC may be functionally connected with metabolic remodeling and immune dysregulation. Liu Q. Y. et al. (2025) further applied shotgun metagenomic sequencing combined with serum and fecal metabolomics to characterize microbiome-based PBC subtypes and functional mechanisms associated with UDCA treatment response. These findings indicate that PBC-associated microbial alterations may not only reflect taxonomic imbalance but may also be linked to bile acid metabolism, amino acid metabolism, inflammatory signaling, and therapeutic responsiveness.

In addition, several studies, including those by Tang et al. (2018), Zhou et al. (2023), and Wang Q. et al. (2024), performed functional pathway analyses based on 16S rRNA gene sequencing-derived prediction. These analyses suggested potential alterations in microbial metabolic pathways; however, such inferred functional profiles should be interpreted cautiously because 16S-based prediction cannot fully capture strain-level functional variation, gene content, or actual metabolite production. Therefore, although the available evidence suggests that gut microbiota dysbiosis in PBC may influence metabolic pathways and immune regulation, the current functional evidence remains fragmented and methodologically heterogeneous. Direct shotgun metagenomics, metatranscriptomics, metabolomics, and host immune profiling are needed to validate these inferred mechanisms and to clarify their clinical relevance.

The clinical relevance of gut microbiota alterations in PBC remains insufficiently defined. Although PBC commonly presents with fatigue, pruritus, cholestatic biochemical abnormalities, and progressive liver injury, available microbiome studies have not consistently evaluated the relationships between gut dysbiosis and these clinical manifestations (Natarajan et al., 2021; Tana and Hirschfield, 2026). Some included studies reported associations between specific microbial taxa and liver biochemical parameters, immune indicators, urinary metabolites, or disease severity-related markers. For example, *Veillonella*, *Klebsiella*, *Neisseria*, *Bifidobacterium*, and *Streptococcus* have been associated with cytokines, bilirubin, urinary metabolites, IgA levels, or cirrhosis-related risk indicators in individual studies (Lv et al., 2016; Tang et al., 2018; Wang Q. et al., 2024; Zang et al., 2025). These findings suggest that gut microbiota profiles may have potential clinical relevance, but the current evidence remains insufficient to determine whether specific microbial signatures are directly associated with symptoms, biochemical disease activity, fibrosis stage, or prognosis. In particular, no included study systematically linked microbial diversity or specific taxa with pruritus severity, fatigue scores, cholestatic enzymes, or validated fibrosis indices, which limits the ability to infer symptom- or severity-specific microbiome signatures.

Treatment-related factors also require further investigation. UDCA remains the first-line therapy for PBC, but a substantial proportion of patients show an inadequate biochemical response (Han et al., 2024). Previous studies have suggested that PBC-associated microbial alterations may be partially restored after UDCA therapy and that gut microbiome–metabolome signatures may be associated with UDCA response (Tang et al., 2018; Liu Q. Y. et al., 2025). Although OCA previously served as a second-line therapeutic option for patients with an inadequate response to UDCA, its withdrawal from the US market has recently been acknowledged by the AASLD, marking an important change in the PBC treatment landscape (American Association for the Study of Liver Diseases, 2025). Therefore, future studies should focus on current and emerging second-line therapies, such as fibrates and PPAR agonists, and evaluate their interactions with gut microbiota, bile acid metabolism, and microbial metabolic activity.

### 4.4 Implication for practice and research

Current evidence indicates that the gut microbiota of patients with PBC undergoes significant alterations in both alpha and beta diversity, as reflected by reduced Shannon index, Chao1 index, and OTUs, together with an increased Simpson index and a relative expansion of dominant taxa. These findings suggest disruption of gut microbial community structure and reduced species richness in PBC. Such diversity changes are accompanied by genus-level dysbiosis, characterized by depletion of SCFA-producing bacteria (e.g., *Faecalibacterium, Blautia, Roseburia,* and *Coprococcus*) and enrichment of potential pathogens or LPS-associated bacteria (e.g., *Neisseria, Klebsiella, Veillonella, Enterococcus,* and *Escherichia*). Collectively, these observations suggest that gut microbial dysbiosis in PBC may influence hepatic immune homeostasis through SCFA deficiency and inflammation-related activation.

From a clinical perspective, gut microbiota profiles may have potential value as adjunctive biomarkers in PBC; however, their clinical application still requires confirmation in large-scale studies, prospective cohorts, and external validation datasets. In addition, given the specific pattern of gut dysbiosis observed in PBC, probiotic supplementation should be applied selectively, with particular emphasis on functional benefits and targeted use in susceptible populations. Because probiotics appear to exert only limited effects on gut microbial diversity in healthy individuals, microbiota-based interventions in patients with PBC should focus more on evidence-based functional outcomes rather than simply aiming to increase microbial diversity.

From a research perspective, future studies should prioritize standardization of methods for assessing alpha diversity, beta diversity, and taxonomic composition, while integrating metagenomics, metabolomics, and transcriptomics to elucidate the functional and clinical implications of genus-level microbial alterations. Although the present meta-analysis identified multiple taxonomic changes at the genus level, the specific roles and mechanisms of individual species within these genera remain insufficiently understood. For example, *Bifidobacterium* and *Lactobacillus* were enriched in patients with PBC, which appears inconsistent with their conventional classification as beneficial probiotics. Future studies should therefore investigate the strain-specific functions and mechanistic roles of these genera in PBC rather than simply categorizing them as “beneficial” or “harmful” bacteria. Further research should also address strain-specific effects, long-term intervention outcomes, and the interactions between the microbiota, bile acid metabolism, and immune regulation, so that microbiome data can be more effectively translated into actionable clinical strategies and precision interventions.

In particular, future studies should incorporate immunological analyses, including microbial antigen profiling, epitope prediction, autoantibody mapping, and T-cell response assays, to determine whether specific gut microbial taxa contribute to PBC through antigenic mimicry or other mechanisms directly related to autoimmune bile duct injury.

### 4.5 Strengths and limitations

This study provides one of the most comprehensive quantitative and qualitative syntheses currently available on gut microbiota alterations in patients with PBC. It included publicly available high-quality studies and covered differences in alpha diversity, beta diversity, and taxonomic composition at the phylum, family, genus, and species levels. To the best of our knowledge, this is the first study to perform both qualitative and quantitative synthesis of the gut microbiota in patients with PBC. In addition, this study was conducted in strict accordance with the PRISMA statement and the methodological recommendations of the Cochrane Collaboration, with comprehensive identification, collection, and analysis of all currently available relevant data.

Several limitations should also be acknowledged. First, most of the included studies were observational in design, and the majority were case–control or cross-sectional studies. Although these studies provide evidence of an association between gut microbiota alterations and PBC, they cannot establish causality or determine whether gut dysbiosis is a cause, consequence, or modifier of disease progression. No randomized controlled trials or well-designed prospective longitudinal studies were available, which limits causal inference and prevents assessment of temporal changes in the gut microbiota during disease development, progression, or treatment. In addition, several included studies had relatively small sample sizes, with some PBC groups including fewer than 20 participants, which may have reduced statistical power, increased uncertainty in effect estimates, and contributed to between-study heterogeneity.

Second, the geographic distribution of the included studies was limited. Most studies were conducted in East Asia, particularly in China and Japan, with limited data from other regions. Because gut microbiota composition may be influenced by ethnicity, diet, environmental exposure, lifestyle, healthcare practice, and medication use, the generalizability of the present findings to broader global populations remains uncertain. Future large-scale, multicenter, and longitudinal studies involving diverse populations are needed to validate whether the microbial signatures identified in this meta-analysis represent universal PBC-associated features or region-specific patterns.

Third, sample type and sequencing methodology varied across the included studies, which may have introduced additional biological and methodological heterogeneity. Most studies analyzed fecal samples, whereas only one study used ileal mucosal samples (Kitahata et al., 2021). Although leave-one-out sensitivity analysis suggested that this mucosal-sample study was not the main driver of the Chao1 result, fecal and mucosa-associated microbiota represent distinct ecological niches and may not be directly comparable. In particular, fecal microbiota mainly reflects luminal microbial communities, whereas mucosa-associated microbiota may be more closely related to local host–microbe interactions, epithelial barrier function, and mucosal immune responses. Because only one study used mucosal samples, we could not perform subgroup analysis to directly compare fecal and mucosa-associated microbial alterations in PBC. In addition, sequencing methods were not fully standardized across studies. Most studies used 16S rRNA gene sequencing, but different hypervariable regions were targeted, including V1–V2 and V3–V4, and different sequencing platforms were applied, including MiSeq, HiSeq, NovaSeq, and HiSeq PE150. These methodological differences may influence taxonomic resolution, relative abundance estimates, detection of low-abundance taxa, and between-study comparability. Although subgroup analysis and meta-regression were performed for sequencing platform where data were available, residual methodological heterogeneity could not be fully excluded.

Fourth, substantial heterogeneity was observed for the Shannon index and ACE index. Although sensitivity analysis, subgroup analysis, and meta-regression were performed, none of these approaches identified the specific sources of the high between-study heterogeneity. Meta-regression showed that country, age, and sequencing platform were not major contributors to the heterogeneity in the Shannon index. However, the scope of meta-regression was limited by the availability of study-level data, and individual-level clinical and treatment-related information was insufficient or inconsistently reported. Consequently, important variables, including BMI, dietary habits, disease duration, clinical manifestations, liver biochemical parameters, fibrosis stage, cirrhosis status, UDCA response, and exposure to antibiotics, probiotics, OCA, fibrates, or other therapies, could not be systematically analyzed. Residual confounding by disease severity, lifestyle factors, and treatment exposure therefore cannot be excluded. Moreover, this limitation prevented us from determining whether specific microbial signatures were associated with symptoms, biochemical activity, disease progression, or therapeutic response.

Fifth, although functional implications were discussed based on the available evidence, functional data across the included studies remained limited and methodologically heterogeneous. Most studies primarily reported microbial composition and diversity, while only a few incorporated direct metagenomic, metabolomic, or immune-factor analyses. Therefore, many functional interpretations, including bile acid metabolism, short-chain fatty acid production, endotoxin-related pathways, tryptophan metabolism, and immune modulation, were inferred indirectly and require further validation through standardized multi-omics and immunological studies. In addition, one study (Liu Q. Y. et al., 2025) further divided patients with PBC into two subgroups while using the same healthy control group, which may have introduced partial non-independence of effect sizes and affected the precision of the pooled estimates.

## 5 Conclusion

This systematic review and meta-analysis demonstrated substantial alterations in the gut microbiota of patients with PBC across multiple levels. Significant changes were observed in both alpha diversity and beta diversity, characterized by reduced Shannon index, Chao1 index, and OTUs, together with an increased Simpson index, indicating marked gut microbial dysbiosis in PBC. These alterations suggest that the gut microbiota may serve as a potential source of biomarkers for PBC, although its clinical value in diagnosis and treatment monitoring requires further validation. Despite the relatively high quality of the available evidence, substantial heterogeneity and regional bias, with most data derived from East Asia, may limit the generalizability of the findings. Overall, this study provides both quantitative and qualitative evidence of gut microbiota alterations in PBC, highlighting the potential role of gut microbial dysbiosis in disease development and progression. Further longitudinal and functional studies are needed to clarify causality and mechanisms and to support the development of microbiota-based precision therapies for PBC.

This figure presents a series of heatmaps illustrating changes in gut microbiota composition at the phylum, family, genus, and species levels across the included studies. The vertical axis represents individual studies, whereas the horizontal axis represents specific microbial taxa at each taxonomic rank. Reported microbial alterations were categorized according to their directional trends as “Increased,” “Decreased,” “Inconsistent,” “NS,” or “NA.” This visualization provides an overall summary of microbial shifts associated with PBC and highlights both consistent and divergent patterns across studies and taxonomic levels.

## Data Availability

The original contributions presented in the study are included in the article/Supplementary material, further inquiries can be directed to the corresponding author.
