# Supplementary material for: Gut microbiota alterations in primary biliary cholangitis: a systematic review and meta-analysis
Source: Front Microbiol. 2026 Jun 10;17:1847865. doi: 10.3389/fmicb.2026.1847865 (PMC13290725; doi:10.3389/fmicb.2026.1847865)
Supplement: Supplementary file 1 [file Table_1.DOCX]

| Databases | Search strategy |
| --- | --- |
| Web of Science (n = 590) | ("primary biliary cholangitis" OR "primary biliary cirrhosis" OR PBC OR "biliary cholangitis") AND ("gut microbiota" OR "intestinal microbiota" OR "gut microbiome" OR "intestinal microbiome" OR "gastrointestinal microbiome" OR "intestinal microflora" OR microbiota OR microbiome OR dysbiosis OR gut OR intestine) |
| PubMed (n = 249) | ("primary biliary cholangitis"[Title/Abstract] OR "primary biliary cirrhosis"[Title/Abstract] OR PBC[Title/Abstract] OR "biliary cholangitis"[Title/Abstract] OR "cholangitis, primary biliary"[MeSH Terms]) AND ("gut microbiota"[Title/Abstract] OR "intestinal microbiota"[Title/Abstract] OR "gut microbiome"[Title/Abstract] OR "intestinal microbiome"[Title/Abstract] OR "gastrointestinal microbiome"[Title/Abstract] OR "intestinal microflora"[Title/Abstract] OR dysbiosis[Title/Abstract] OR "microbiota"[MeSH Terms] OR "microbiome"[MeSH Terms] OR "gut"[Title/Abstract] OR "intestine"[Title/Abstract]) |
| Embase (n = 459) | ('gut microbiota' OR 'intestinal microbiota' OR 'gut microbiome' OR 'intestinal microbiome' OR 'gastrointestinal microbiome' OR microbiota OR microbiome OR dysbiosis OR 'intestinal microflora') AND ('primary biliary cholangitis' OR 'primary biliary cirrhosis' OR pbc OR 'biliary cholangitis') |
| Cochrane Library (n = 26) | ("primary biliary cholangitis" OR "primary biliary cirrhosis" OR PBC OR "biliary cholangitis") AND ("gut microbiota" OR "intestinal microbiota" OR "gut microbiome" OR "intestinal microbiome" OR "gastrointestinal microbiome" OR "intestinal microflora" OR microbiota OR microbiome OR dysbiosis OR gut OR intestine) |

**Table S1. Search strategy in PubMed, Embase, Web of Science** **databases and Cochrane Library. All Databases Search deadline:** **2026-01-23**

**Table S2. Methodology and findings of the included studies assessing Alpha diversity**

| Study | Analysis | Finding |
| --- | --- | --- |
| Lv et al. | Chao1, Shannon | no sig. difference |
| Zang et al. | Shannon | higher |
| Furukawa et al | observed_OTUs  Shannon–Wiener indices  PD_whole_tree | sig. difference  higher  no sig. difference |
| Tang et al. | observed_OTUs  Shannon | lower  no sig. difference |
| Zhou et al. | Ace  Chao1  observed features | lower  lower  lower |
| Liu et al.  Clostridia low  Clostridia higher | Shannon index | no sig. difference  high |
| Kitahata et al. | Chao-1 index,  Shannon index | lower  no sig. difference |
| Wang et al.2024 | Chao，Ace, Sobs, Shannon,  Simpson, Coverage | sig. difference  no sig. difference |
| Wang et al.2021 | Ace, Chao, Simpson, Shannon | no sig. difference |
| Ding et al. | Chao1, Shannon | sig. difference |

**Table S3. Methodology and findings of the included studies assessing beta diversity**

| Study | Metric | Analysis | Finding |
| --- | --- | --- | --- |
| Lv et al. | unweighted UniFrac  euclidean or weighted | PCoA， PERMANOVA | no sig. difference |
| Zang et al. | weighted UniFrac | PCoA | sig. difference |
| Furukawa et al. | weighted Unifrac | PCoA | sig. difference |
| Tang et al. | unweighted UniFrac | PCoA, PERMANOVA | sig. difference |
| Zhou et al. | weighted UniFrac | PCoA | sig. difference |
| Liu et al. | aitchison distance | PCoA, PERMANOVA | sig. difference |
| Kitahata et al. | unweighted UniFrac | PERMANOVA | no sig. difference |
| Wang et al. (2024) | weighted Unifrac | PCoA | sig. difference |
| Wang et al. (2021) | NA | NA | NA |
| Ding et al. | NA | NA | NA |

1. Level: Order


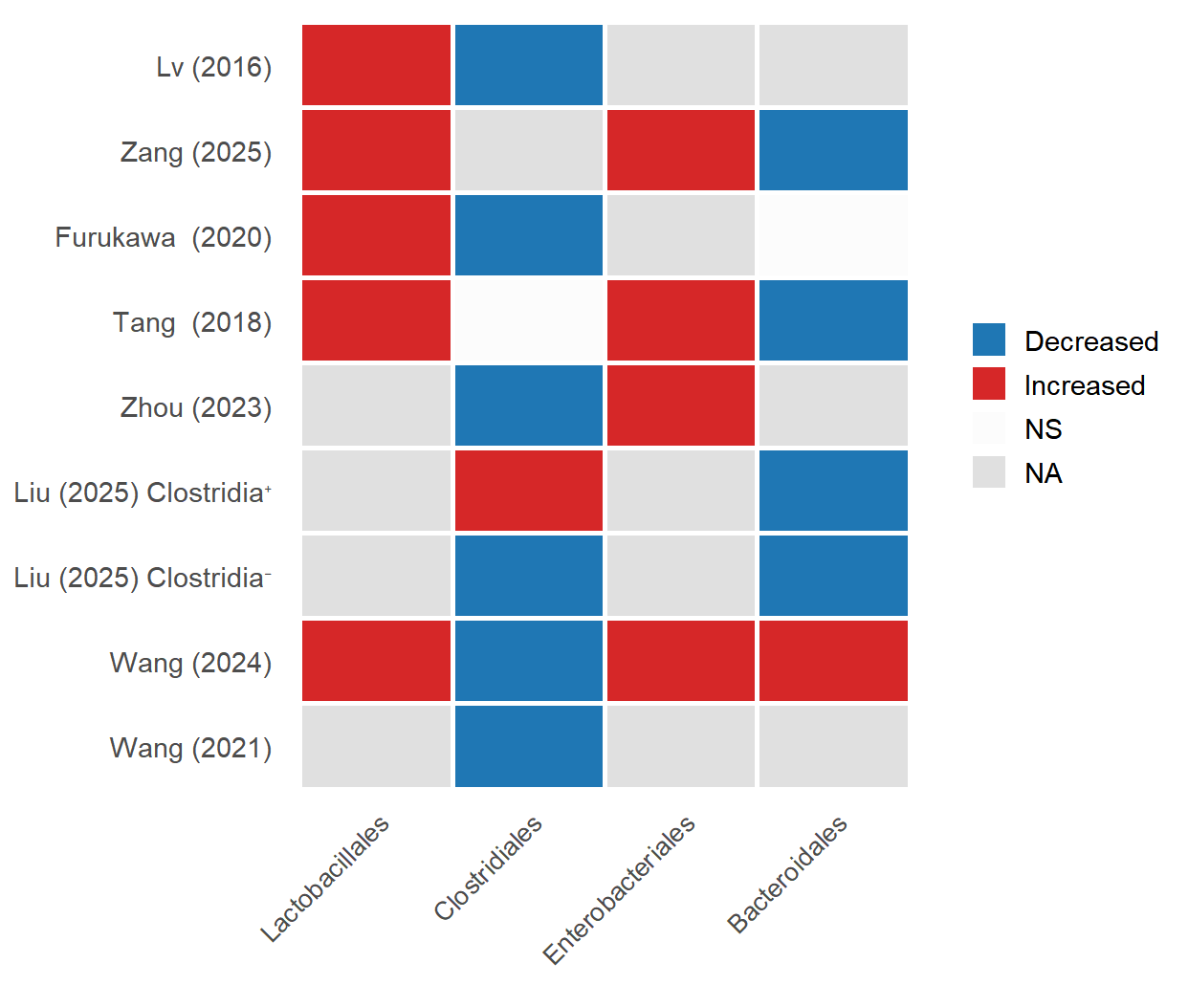


1. Level:
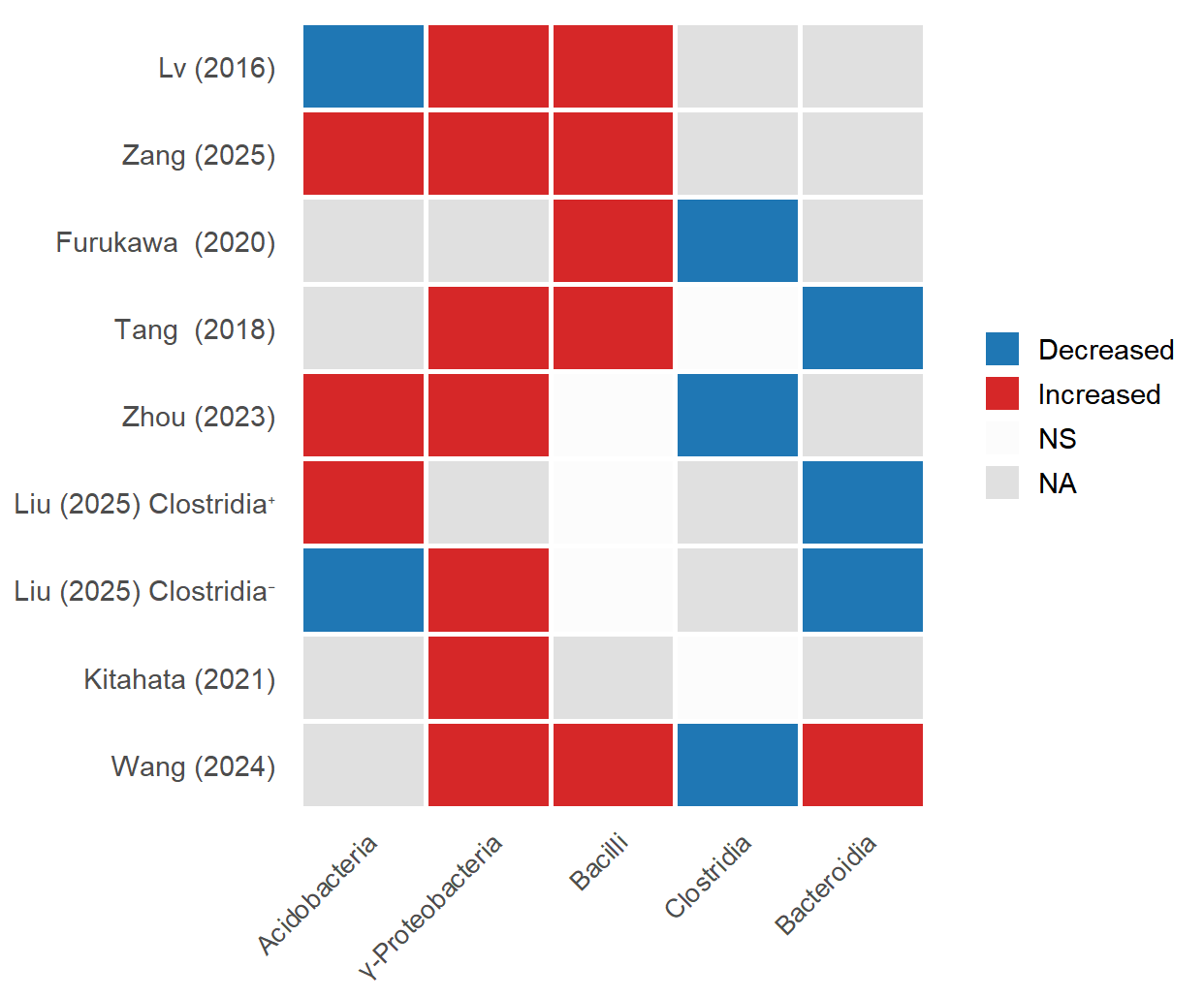
 Class

**FIGURE S1.** Changes in gut microbiota composition in PBC across multiple taxonomic levels.
